# TNF*α* and IFN*γ* rapidly activate PI3K-AKT signaling to drive glycolysis that confers mesenchymal stem cells enhanced anti-inflammatory property

**DOI:** 10.1186/s13287-022-03178-3

**Published:** 2022-10-04

**Authors:** Chenchang Xu, Chao Feng, Peiqing Huang, Yinghong Li, Rui Liu, Chunxiao Liu, Yuyi Han, Lei Chen, Yayun Ding, Changshun Shao, Yufang Shi

**Affiliations:** 1 The First Affiliated Hospital of Soochow University, Institutes for Translational Medicine, State Key Laboratory of Radiation Medicine and Protection, Suzhou Medical College of Soochow University, Suzhou, Jiangsu China; 2 Department of Interventional Radiology and Vascular Surgery, The Affiliated Suzhou Hospital of Nanjing Medical University, Suzhou, Jiangsu China

**Keywords:** MSCs, IBD, Glycolysis, PI3K-AKT, Anti-inflammation

## Abstract

**Background:**

Mesenchymal stem/stromal cells (MSCs) acquire immunosuppressive capacity only in an inflammatory microenvironment. This can be recapitulated in vitro by treating MSCs with inflammatory cytokines TNF*α* and IFN*γ*, which induce indoleamine 2,3-dioxygenase (IDO) and TNF-stimulated gene-6 (TSG-6). However, the signaling pathways downstream of the cytokines remain to be elucidated.

**Methods:**

Inflammatory bowel disease (IBD) mouse model was established by subjecting mice to dextran sulfate sodium (DSS) in drinking water for 7 days. Human UC-MSCs were pretreated with TNF-*α* and IFN-*γ* for 24 h and were then infused intravenously at day 2 of DSS administration. Colon tissues were collected for length measurement and histopathological examination. The serum level of IL-6 in mice was measured by enzyme-linked immunosorbent assay. Real-time PCR and Western blot were used to examine the mRNA level and protein expression. MSCs overexpressing constitutive active AKT or dominant negative AKT were generated and were analyzed. The glycolysis level of the MSCs was measured using Extracellular Flux Analyzer. 2-NBDG was used to monitor the uptake of glucose by MSCs.

**Results:**

TNF*α* and IFN*γ* treatment led to rapid consumption of glucose and metabolic skewing toward glycolysis in MSCs, which was required for the therapeutic efficacy of MSCs on IBD. Blockade of glycolysis in MSCs inhibited the expression of immunomodulatory molecules, IDO and TSG-6, as well as the therapeutic effect on IBD. Moreover, PI3K-AKT signaling axis was rapidly activated and was required for the skewing toward glycolysis induced by TNF*α* and IFN*γ*. MSCs expressing dominant negative AKT were compromised in their therapeutic efficacy on IBD.

**Conclusion:**

The glycolysis-dependent anti-inflammatory property of MSCs conferred by inflammatory cytokines is mediated by PI3K-AKT signaling pathway.

**Supplementary Information:**

The online version contains supplementary material available at 10.1186/s13287-022-03178-3.

## Background

Mesenchymal stem cells (MSCs) are a heterogeneous group of stromal cells that play important roles in organ architecture, tissue homeostasis, and repair. MSCs expanded in vitro have been widely used for the therapy of autoimmune and inflammatory diseases. MSCs were reported to successfully treat patients with severe, non-drug-responsive inflammatory diseases such as acute GvHD [1], Crohn’s disease, and systemic lupus erythematosus. Recent studies show that MSCs-based therapy modulates inflammation and ‘empowers’ other cells to facilitate tissue regenerations, rather than to replace the damaged cells [2, 3]. The immunomodulatory property of MSCs is induced or licensed by inflammatory factors such as TNF*α*, IFN*γ*, and IL1. Many studies demonstrated that human MSCs stimulated by inflammatory factors TNF*α* and IFN*γ* exert their anti-inflammatory effects via IDO, TSG-6, and PGE2. Genetic knockdown or chemical inhibition of IDO abolishes the anti-inflammatory function of human MSCs. TSG-6, which is secreted from stimulated MSCs and binds to hyaluronan, reduces inflammation in mouse models of arthritis [4], myocardial infarction [5, 6], corneal injury [6], acute lung injury [7], and peritonitis [8].

It was reported that glycolysis is required for MSCs to acquire the potent anti-inflammatory property in response to pro-inflammatory cytokines [9], although the metabolic reprogramming of MSCs associated with the anti-inflammatory function remains poorly understood. We previously demonstrated that TNF*α* and IFN*γ* are able to confer MSCs potent therapeutic efficacy in several inflammatory diseases such as lipopolysaccharide (LPS)-induced acute lung injury (ALI) [7] and EAE [10–12], the signaling pathways downstream of the inflammatory cytokines remains to be elucidated.

We tested the role of glycolysis for the anti-inflammatory effect of MSCs and found that the glycolytic level increased within several minutes after MSCs were treated by TNF*α* and IFN*γ*. This process was caused by acutely activated PI3K-AKT signaling, which subsequently augments glucose uptake and promotes HKII enzyme activity. More importantly, these acute activation processes of PI3K-AKT signaling were critical for the production of anti-inflammation factors such as IDO1 and TSG-6 and anti-inflammatory therapeutic effect in the inflammatory bowel disease (IBD) model. Our study indicates that the glycolysis augmentation in MSCs is a fast response to TNF*α* and IFN*γ*. PI3K-AKT signaling is rapidly activated downstream TNF*α* and IFN*γ* to promote glycolysis. This finding has implications in MSCs-based anti-inflammation therapy.

## Materials and methods

### Cell culture of MSCs

Human UC-MSCs were provided by Wuxi Sinotide New Drug Discovery Institutes (Wuxi, China). Their identity was confirmed by cell surface markers including CD105(+), CD73(+), CD29(+), CD90(+), CD44(+), CD45(−), CD31(−), CD34(−), and HLA-DR(−). They were maintained in low-glucose DMEM (HyClone, USA), supplemented with FBS (10%) (Gibco, USA), penicillin/streptomycin (10 units) (ThermoFisher, USA), 10 ng/ml human-bFGF (R&D, USA), and at 37 °C in the presence of 5% CO_2_. The media were replaced for every 72 h and the cells were split twice a week. Cells were used before the 12th passage.

### RNA preparation and qRT-PCR

Total RNA was extracted from cell lysates according to the manufacturer’s instructions using Trizol reagent (Thermo Fisher Scientific, USA). First-strand cDNA synthesis was performed using PrimeScript™ RT Master Mix (TaKaRa Biotech, Dalian, China) according to the manufacturer’s instructions. Relative mRNA levels were quantified by qPCR using ABI SYBR Green on QuantStudio 6 Flex (Applied Biosystems, MA, USA). Target genes were normalized to β-actin. Primer sequences are listed in Additional file 1: Table S1.

### Western blot

Proteins were extracted from cells using lysis buffer (50 mM Tris–HCl, 150 mM NaCl, pH = 7.4, 1% Triton X-100, 1% SDS) supplemented with protease inhibitors (Roche) and 1 mM PMSF (Sigma-Aldrich) and then separated by SDS-PAGE and transferred to polyvinylidene difluoride membranes (Merck). Antibodies used in the present study include IDO1 (GeneTex, GTX634652), HA (Abcam), COX2 (Cell Signaling Technology), AKT (Cell Signaling Technology, 4691), Phospho-AKT (S473) (Cell Signaling Technology, 4060), HKII (Cell Signaling Technology, 2867), β-actin (Cell Signaling Technology, 3700), GLUT1 (ThermoFisher Scientific, PA1-46,152), and GAPDH (Cell Signaling Technology, 5174) were brought from Cell Signaling Technology.

### ELISA

The TSG-6 protein level in the medium of human MSCs cultured with inflammatory cytokines was measured by ELISA as previously described [17]. Briefly, a 96-well high binding plate was coated with 50 μl of 10 μg/ml TSG-6 antibody (clone A38.1.20, Santa Cruz Biotechnology) overnight, in a coating buffer of 0.2 M sodium bicarbonate (pH 9.2). The plate was washed with PBS, blocked with blocking buffer (0.25% BSA and 0.05% Tween 20 in PBS) for 30 min. Samples (100 μl) and a standard of human recombinant TSG-6 protein (R&D Systems) in blocking buffer were added and incubated at room temperature for 2 h. Then, the plate was washed with PBST (0.05% Tween 20) three times, followed by the addition of a biotinylated anti-human TSG-6 antibody (50 μl of 0.5 μg/ml; R&D Systems) and incubated for 2 h at room temperature. After washing with PBST three times, streptavidin–HRP (50 μl; R&D Systems) was added for 30 min at room temperature and developed using TMB substrate (Cell Signaling Technology).

### Flow cytometry

Cells were made to single cell suspensions by trypsin treatment. For the GLUT1 analysis, cells were fixed by a fixation/permeabilization kit (Invitrogen, 00-5521-00) at room temperature for 30 min. After the fixation and permeabilization, cells were stained by GLUT1 antibody (Thermo Fisher, PA1-46,152) in the presence of blocking solution (1% BSA in PBS) overnight. Cells were incubated with fluorochrome-coupled secondary antibody (Abcam, ab150075) at room temperature for 1 h. After PBS washing, cells were recorded using a CytoFLEX (BECKMAN COULTER, Inc.) and analyzed with FlowJo Version 10.1 software (TreeStar, Ashland, OR).

### Extracellular acidification rate

The glycolytic function of human UC-MSCs was analyzed by XFe24 Extracellular Flux Analyzer (Seahorse Bioscience). Human UC-MSCs were seeded on a 0.1% gelatin coated XFe 24 plate. The cells were treated with TNF*α* and IFN*γ* or inhibitors before the measuring for the indicated time. Extracellular acidification rate (ECAR) was measured using the glycolysis stress test kit in Seahorse XF Base Medium as described in the manufacturer’s instructions. The results were analyzed using Wave software (Seahorse/Agilent).

### 2-NBDG uptake assay

The fluorescent D-glucose analog 2-(N-(7-nitrobenz-2-oxa-1,3-diazol-4-yl)amino)-2- deoxyglucose (2-NBDG) (ThermoFisher Scientific, N13195) was used as a fluorescent indicator to evaluate glucose uptake. The MSCs were grown in 6-well plates at 37 °C with 5% CO_2_. When the cells reached 90% confluence, 2-NBDG was added to the cells to achieve concentrations of 5 mg/ml in the medium. TNF*α* and IFN*γ* were added at the same time with 2-NBDG. The inhibitors were treated 2 h before 2-NBDG addition. The viruses of Myr-AKT and DN-AKT were added 72 h before 2-NBDG addition. The experiment was performed in triplicate. The cells were incubated for 1 h with the probe, then washed with cold PBS twice. Then the cells were collected to measure the 2-NBDG signal by flow cytometry (BECKMAN COULTER, Inc.).

### Hexokinase activity assay

Hexokinase activity was assayed in MSCs using the Hexokinase activity kit (MAK091, Sigma, St. Louis, MO, USA) according to the manufacturer's instructions. The MSCs were cultured in a 24-well plate. The cells were treated by TNF*α* and IFN*γ* for different time periods. MSCs were treated with inhibitors LY294002 (10 μM, MCE, USA) or triciribine (50 μM, MCE, USA) before addition of TNF*α* and IFN*γ*. The viruses of Myr-AKT and DN-AKT were added 72 h before addition of TNF*α* and IFN*γ*. The results were normalized to the amount of protein in the sample.

### IBD mouse model

To induce experimental colitis, 8-week-old C57BL/6 J mice were administrated with 4% DSS (36,000–50,000 MW, MP Biomedicals) in autoclaved drinking water for 7 days.

TNF*α* and IFN*γ* treated human UC-MSCs (1 × 10^6^) were intravenously injected to treat IBD mice on day 2 after the beginning of DSS treatment. Control group mice received normal drinking water. The body weight of each mouse was monitored daily during the IBD induction process. The serum and the colon samples were collected for further processing. All experimental mice were sacrificed at the end of the 7-day DSS treatment. The procedures were approved and conducted under a protocol approved by Ethics Committee of Soochow University.

### Histological analysis

Colon tissues were fixed in 4% paraformaldehyde, paraffin-embedded, and processed for histological analysis. Colon sections with a 5-μm thickness were subjected to hematoxylin and eosin (H&E) staining and examined by light microscopy. The severity of IBD symptoms was evaluated by scoring the extent of bowel wall thickening (grades, 0–3: 0, none; 1, mucosa; 2, mucosa and submucosa; 3, transmural), the damage of crypt (grades, 0–3: 0, none; 1, loss of goblet cells; 2, only surface epithelium intact; 3, loss of entire crypt and epithelium), and the infiltration of inflammatory cells (grades, 0–2: 0, none; 1, mild to moderate; 2, severe).

### Statistical analysis

Data were analyzed with Graphpad Prism software (version 8). Data values are presented as means ± SEM. Differences were considered significant when *p* values were below 0.05. For a two-group comparison, two-tailed unpaired *t* tests were performed. For multiple group comparison, a one-way analysis of variance test was performed.

## Results

### Glycolysis is required for MSCs to exert their therapeutic effect in inflammatory bowel diseases mouse model

**Fig. 1:**
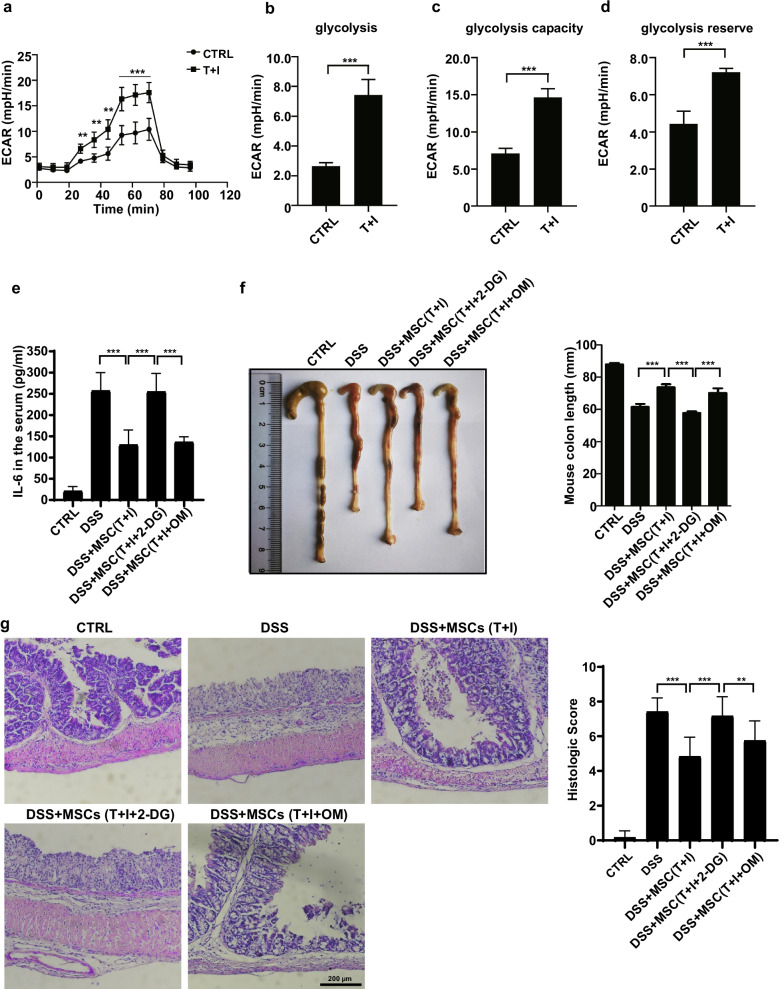
TNF*α*- and IFN*γ*-induced glycolysis is required for the therapeutic effect of hUC-MSCs on IBD. **a** Extracellular acidification rate (ECAR) of hUC-MSCs treated with or without TNF*α* and IFN*γ* for 24 h (TNF*α* and IFN*γ*, 10 ng/ml each; *n* = 4 for each group; repeated three times). ECAR was measured under basal conditions, in response to 10 mM glucose (= basal glycolysis), and upon blocking the mitochondrial ATP generation by 1 µM oligomycin. Compensatory effects on ECAR following interference with oxidative phosphorylation (OXPHOS) represent the maximal glycolysis capacity. **b** The basal and **c** maximal glycolysis values in figure (**a**) were statistically analyzed. **d** Calculation of the glycolysis reserve is subtraction basal glycolysis from maximal glycolysis. **e** The IL-6 protein levels in the serum were examined by ELISA. **f** The colon length of IBD mice treated with hUC-MSCs. The right panel shows the statistic of the colon length of IBD mice. **g** The representative H&E stained colon sections of IBD mice treated with the hUC-MSCs and their histological scores. Scale bar, 200 μm. Data are presented as mean ± SEM. ***, *p* < 0.001. *n* = 5 for each groups

TNF*α* and IFN*γ*-licensed human MSCs have been proved to possess a powerful anti-inflammatory effect in numerous studies [12–14]. To investigate the role of glycolysis in MSCs cell therapies, we firstly examined the real-time changes in the rate of extracellular acidification (ECAR), as a representation of glycolysis level. We observed the enhanced ECAR (i.e., basal glycolysis, glycolytic capacity, and glycolytic reserve) after TNF*α* and IFN*γ* treating MSCs for 24 h (Fig. 1a–d). This result is consistent with the report that the MSCs licensing by inflammatory factors requires glycolysis [9]. Next, we tested the function of TNF*α*- and IFN*γ*-licensed MSCs by employing dextran sulfate sodium (DSS)-induced inflammatory bowel diseases (IBD) model in C57BL/6 J mice. We found a single intravenous injection of TNF*α* and IFN*γ*-licensed MSCs significantly reduced the inflammatory parameters in IBD mice (Fig. 1e, f). Surprisingly, the therapeutic effects of MSCs were abolished when they were treated with 2-Deoxy-D-glucose (2-DG). However, there is no difference between oligomycin (OM)-treated T + I-MSCs and T + I-MSCs. This indicates that the glycolysis reprogramming, but not oxidative phosphorylation (OXPHOS), in MSCs is required for the licensing by TNF*α* and IFN*γ* (Fig. 1e, f). The histology images of colons are consistent with inflammatory parameters (Fig. 1g). In conclusion, the MSCs licensing by TNF*α* and IFN*γ* requires glycolysis.

### Expression of immunosuppressive effectors in MSCs relies on glycolysis

**Fig. 2:**
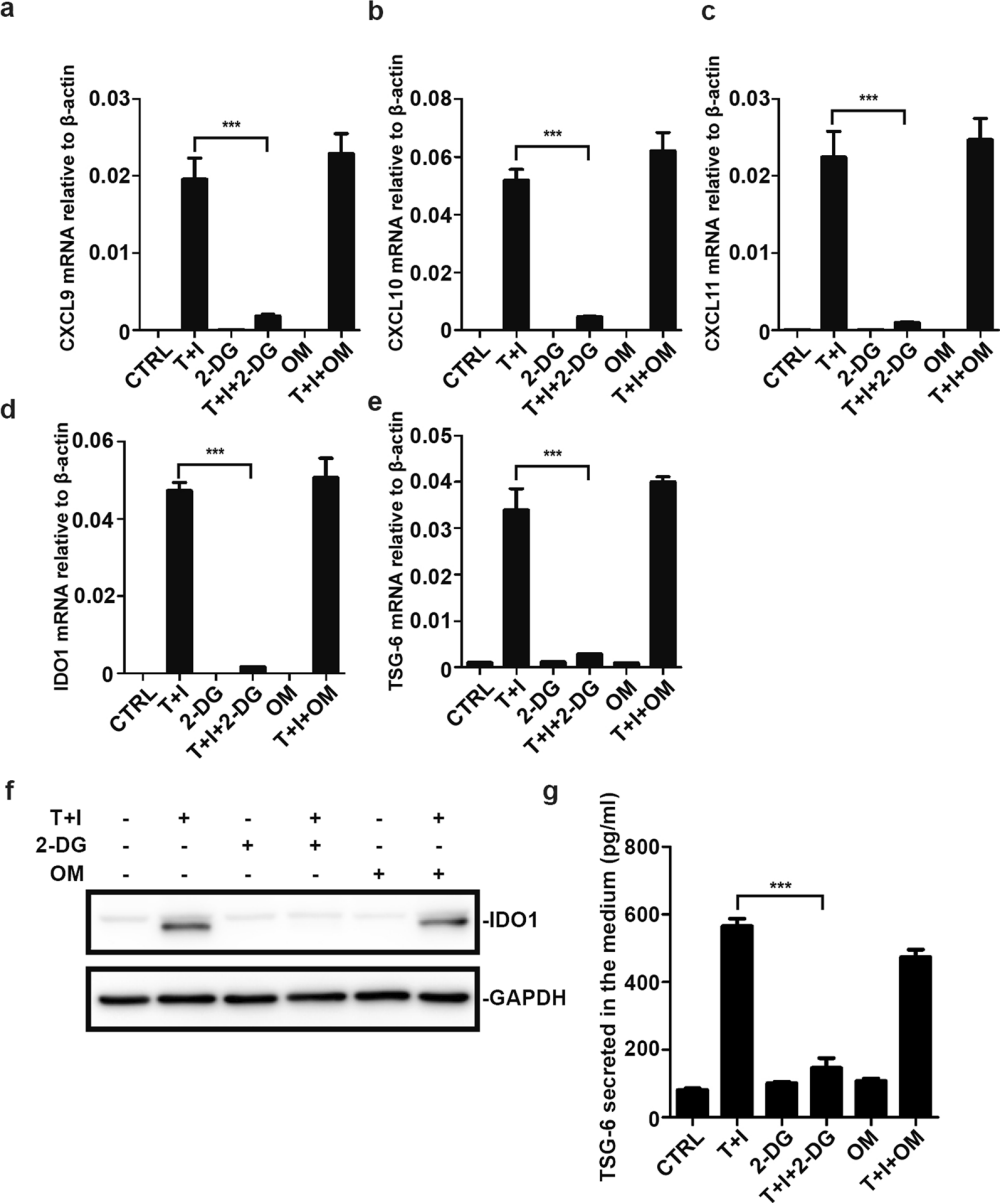
Blocking glycolysis of hUC-MSCs abolishes the expression of cytokines and anti-inflammatory factors. **a**–**e** CXCL9, CXCL10, CXCL11, IDO1, and TSG-6 mRNA levels of MSCs were measured. The MSCs were treated with TNF*α* and IFN*γ* (10 ng/ml each) with or without 2-Deoxy-D-glucose (2-DG) and oligomycin (OM) for 24 h. All sample transcript levels were normalized to the level of β-actin. MSCs were treated as in (**a**), then the cells were collected and the IDO1 protein level (**f**) was examined by western blotting. The TSG-6 protein level (**g**) in the medium was examined by ELISA. Data are presented as mean ± SEM. of triplicates (**a**, **c**) ***, *p* < 0.001

Inflammatory factors TNF*α* and IFN*γ* licensed MSCs are known to produce many types of effector factors including IDO, TSG-6, and other cytokines. This was confirmed in our experiments (Fig. 2a–c). Among these factors, IDO and TSG-6 were critical for ameliorating inflammatory processes such as acute lung injury (ALI) [7]. Therefore, we examined whether the expression of IDO and TSG-6 was affected by glycolysis. We checked the expression profile of hUC-MSCs after glycolysis inhibitor (2-DG) treatment. Both mRNA and protein levels of IDO and TSG-6 were dramatically inhibited in 2-DG treated MSCs, their expression was not affected by the inhibition of OXPHOS (Fig. 2a–c). The mRNA level of CXCL9, CXCL10, and CXCL11 was compromised by the 2-DG. In brief, TNF*α* and IFN*γ* induced upregulated expression of IDO and TSG-6 in a manner dependent on glycolysis reprogramming. These findings were consistent with the results obtained with the IBD mouse model.

### PI3K-AKT signaling is acutely activated by TNF*α* and IFN*γ* and is essential for the expression of IDO and TSG-6

**Fig. 3:**
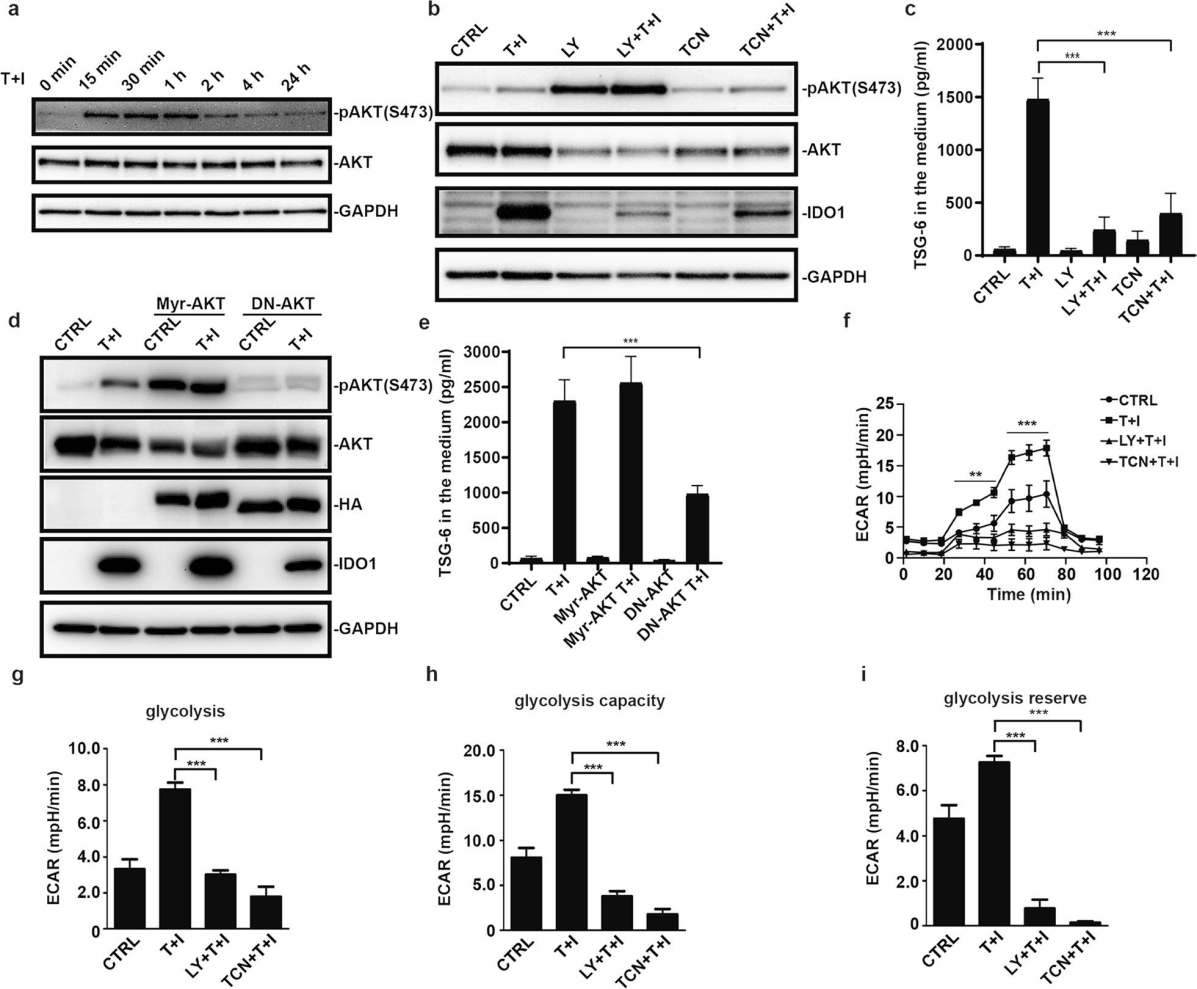
TNF*α* and IFN*γ* acutely increase the phosphorylation of AKT. **a** MSCs were stimulated with TNF*α* and IFN*γ* (10 ng/ml each) for different periods and lysed. Total AKT and phosphorylated AKT (S473) were examined by western blotting. **b** MSCs were pretreated with LY294002 (LY) or triciribine (TCN) for 1 h and then TNF*α* and IFN*γ* were added into the medium for 24 h. MSCs were lysed and total protein was extracted. IDO1, total AKT, and phosphorylated AKT (S473) were examined by western blotting. **c** MSCs treated as in (**b**), and TSG-6 in the medium were examined by ELISA. **d** MSCs were infected by constitutive active form Myr-AKT or dominant-negative AKT (DN-AKT) adenovirus two days before TNF*α* and IFN*γ* stimulation. After TNF*α* and IFN*γ* treatment for 24 h, MSCs were lysed and total protein was extracted. IDO1, total AKT, and phosphorylated AKT (S473) were examined by western blotting. HA-tagged Myr-AKT or HA-tagged DN-AKT was immunoblotted by antibody against HA. **e** MSCs treated as in (**d**), and TSG-6 in the medium were examined by ELISA. **f** Extracellular acidification rate (ECAR) of hUC-MSCs was monitored when TNF*α* and IFN*γ* treatment for 1 h. **g** The basal and (**h**) maximal glycolysis values in figure (**f**) were statistically analyzed. (**i**) Calculation of the glycolysis reserve in figure (**f**) is subtraction basal glycolysis from maximal glycolysis. Data are presented as mean ± SEM. of triplicates (**d**–**i**) ***, *p* < 0.001

PI3Ks are recognized for their roles as second messengers regulating multiple key cellular functions [15, 16]. Given that the activation of PI3K is required for IFN-*γ* induced-IDO expression [17] and Toll-like receptor-induced changes in glycolytic metabolism regulate dendritic cell activation is AKT dependent [18, 19], we reasoned that AKT would be essential for the increased glycolysis and the induction of IDO and TSG-6 expression. We found that TNF*α* and IFN*γ* stimulation increased the phosphorylation of AKT rapidly at the Ser473 site, which is necessary for AKT activation. After 15 min of stimulation by TNF*α* and IFN*γ*, the pAKT already reached the highest level (Fig. 3a). This rapid response of AKT signaling suggested that AKT may act upstream of IDO and TSG-6. ERK1/2 signaling, on the other hand, is not changed upon TNF*α* and IFN*γ* stimulation (data not shown). We next examined whether the expression of IDO and TSG-6 relied on the AKT activation. We used two PI3K-AKT signaling inhibitors, LY294002 (LY) and triciribine (TCN), to block the activation of AKT. Consistent with the proposed role for AKT in the expression of downstream genes, the two distinct AKT inhibitors substantially blunted the expression of IDO and TSG-6 (Fig. 3b, c). Unexpectedly, treatment of hUC-MSCs with LY294002 for 24 h enhanced AKT phosphorylation specifically. This may be because of the redundancy of other kinases which could phosphorylate AKT. Next, we employed the constitutive active form of AKT (Myr-AKT) and the dominant-negative form of AKT (DN-AKT) to confirm the effect of PI3K-AKT signaling. Our results showed that the kinase-dead form of AKT (DN-AKT) decreased IDO and TSG-6 protein level compared to the control group when MSCs were treated with TNF*α* and IFN*γ*. Nevertheless, the constitutive active form of AKT (Myr-AKT) did not further boost IDO and TSG-6 level (Fig. 3d, e). Also, Myr-AKT alone is not sufficient to induce the expression of IDO and TSG-6. Furthermore, we examined the real-time changes in the ECAR of MSCs after the treatment with PI3K-AKT inhibitors. Acute activation of PI-3 K-AKT signaling was found to be required for the increased ECAR in hUC-MSCs (Fig. 3f–i). Together these data indicate that the acutely activated PI3K-AKT signaling in response to TNF*α* and IFN*γ* drives the enhanced expression of IDO and TSG-6 by promoting glycolysis.

### PI3K-AKT signaling increases the glucose uptake in TNF*α*- and IFN*γ*-treated MSCs

**Fig. 4:**
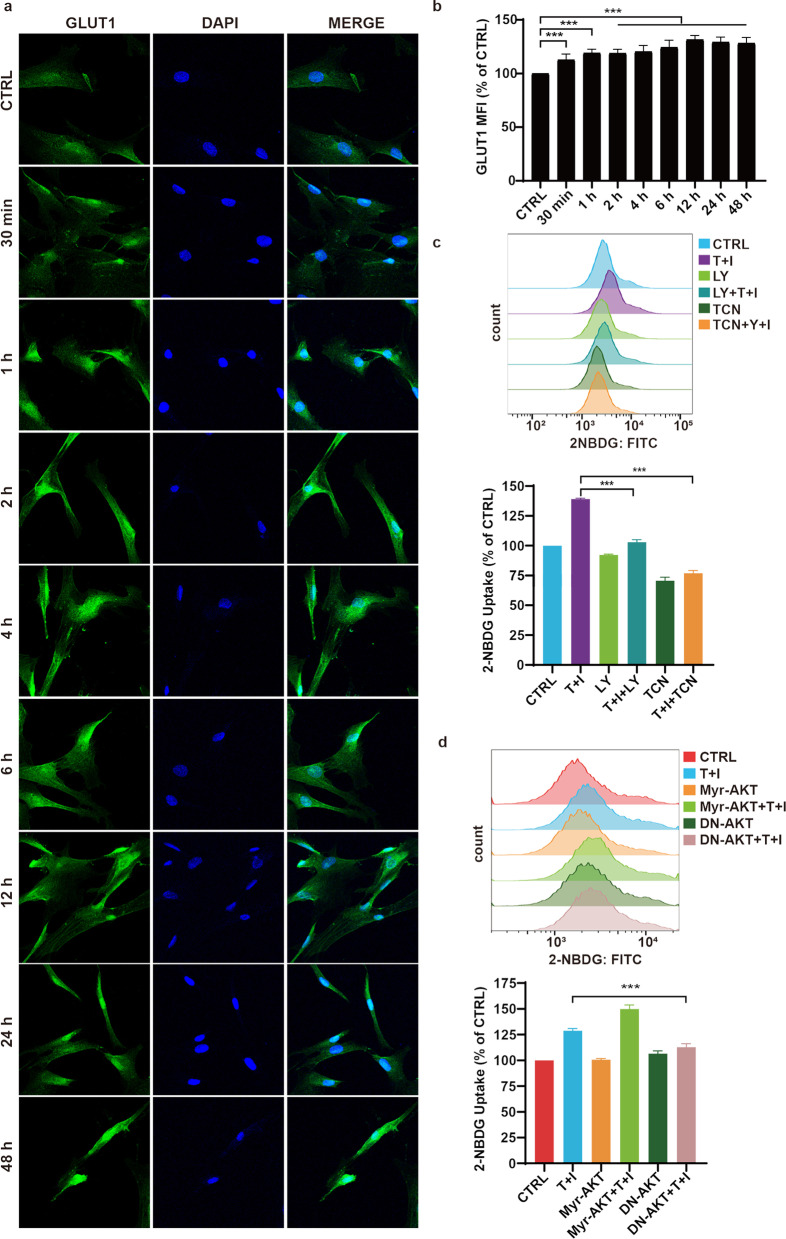
Enhanced glucose uptake relies on the PI3K-AKT signaling. **s** Immunofluorescence staining of GLUT1 in MSCs. MSCs were stimulated by TNF*α* and IFN*γ* for different periods as noted. **b** GLUT1 expression was quantified by flow cytometry (*n* = 3) based on the median fluorescence index (MFI). MSCs were stimulated by TNF*α* and IFN*γ* for different periods as noted. **c**, **d** The uptake of 2-NBDG is measured by flow cytometry. MSCs were pretreated with LY294002 (LY) or triciribine (TCN) for 1 h and then TNF*α* and IFN*γ* were added into the medium for 1 h. **d** The uptake of 2-NBDG is measured by flow cytometry. MSCs were infected by Myr-AKT or dominant-negative AKT (DN-AKT) adenovirus two days before TNF*α* and IFN*γ* stimulation. Then MSCs were treated with TNF*α* and IFN*γ* for 1 h. Scale bar, 100 μm. Data are presented as mean ± SEM. of triplicates (**b**–**d**) ***, *p* < 0.001

To investigate the mechanism by which PI3K-AKT signaling upregulates glycolysis, we firstly focused on the glucose uptake by MSCs. Glucose transporter 1 (GLUT1) is responsible for the uptake of glucose into the cells. We found the expression of GLUT1 on the cell surface were quickly upregulated and persisted at a high level until at least 48 h after treatment by TNF*α* and IFN*γ* (Fig. 4a, b). We used 2-NBDG, a fluorescent glucose analog, to monitor the uptake of glucose in MSCs at different time points after TNF*α* and IFN*γ* stimulation. TNF*α* and IFN*γ* enhanced the influx of glucose in MSCs at a very early time (Fig. 4c, d), which corresponds to the high levels of glycolysis. To confirm the role of PI3K-AKT signaling in regulating glucose uptake, we blocked PI3K-AKT signaling by using inhibitors or by introducing dominant-negative mutant. The data showed that blocking of PI3K-AKT signaling reduced the glucose influx (Fig. 4c, d). Based on flow cytometry of 2-NBDG, inhibition of PI3K-AKT signaling did not completely abolish the uptake of glucose. This means that a basal level of glucose influx is required for the survival of MSCs. Activated PI3K-AKT signaling may account for the enhanced fraction of glucose influx. The basal levels of glucose influx and glycolysis in hUC-MSCs are not at very high level (Fig. 4c, 1a, 3f). Therefore, 35% of the glucose influx augment had already changed the glucose metabolism significantly.

### HKII activity is increased upon TNF*α* and IFN*γ* stimulation

**Fig. 5:**
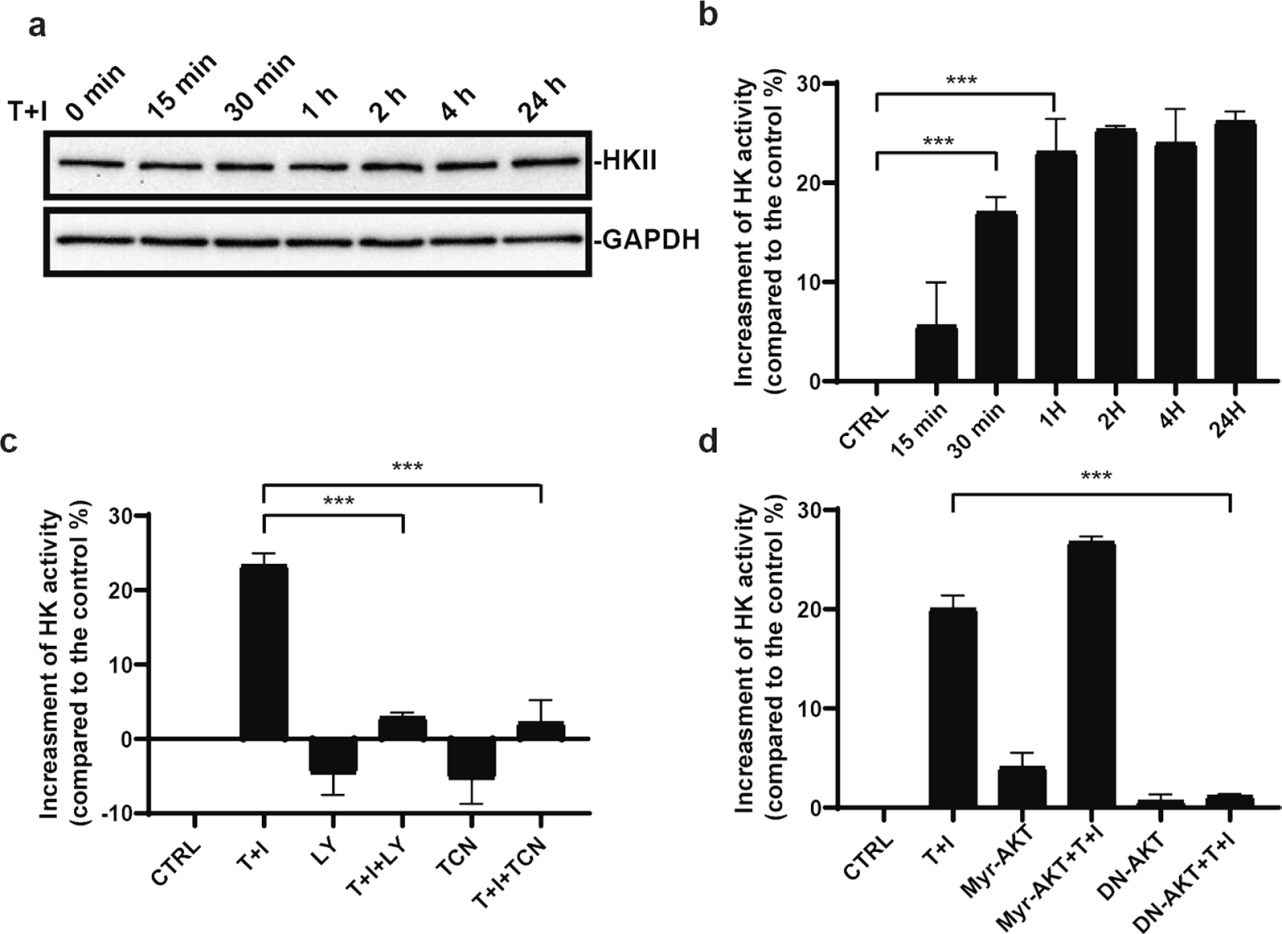
PI3K-AKT signaling is required for the increase of HKII activity. **a** The protein level of HKII is not changed upon TNF*α* and IFN*γ* stimulation. **b** HKII activity is increased quickly after TNF*α* and IFN*γ* stimulated. **c** MSCs were pretreated with LY294002 (LY) or triciribine (TCN) for 1 h and then TNF*α* and IFN*γ* were added into the medium for 1 h. HKII activity of MSCs was analyzed by hexokinase activity assay. **d** MSCs were infected by adenovirus expressing constitutive active form Myr-AKT or dominant-negative AKT (DN-AKT) two days before TNF*α* and IFN*γ* stimulation. After TNF*α* and IFN*γ* treatment for 1 h, HKII activity of MSCs was analyzed by hexokinase activity assay. Data are presented as mean ± SEM. of triplicates (**b**–**d**) ***, *p* < 0.001

Hexokinase II (HKII) catalyzes ATP-dependent phosphorylation of glucose to produce glucose 6-phosphate, which is the first main rate-limiting enzyme of glycolysis [20–23]. We next investigated whether HKII was regulated by TNF*α* and IFN*γ*. Surprisingly, during the TNF*α* and IFN*γ* treatment, the HKII protein level remained unchanged (Fig. 5a). Next, the enzyme activity of HKII was measured. We found that the HKII enzyme activity was quickly increased after the TNF*α* and IFN*γ* treatment (Fig. 5b). This indicated that the acutely activated PI3K-AKT signaling could regulate the glycolysis through modifying HKII. The inhibitors of PI3K-AKT signaling abolished the increase of HKII activity (Fig. 5c). To confirm the results shown above, we also used dominant-negative AKT to block the activation of AKT. HKII activity was indeed blocked by the dominant-negative AKT (Fig. 5d). Thus, HKII is the key enzyme in regulating the anti-inflammation function of MSCs.

### TNF*α*- and IFN*γ*-activated PI3K-AKT signaling is indispensable for the therapeutic effect of MSCs on IBD

**Fig. 6:**
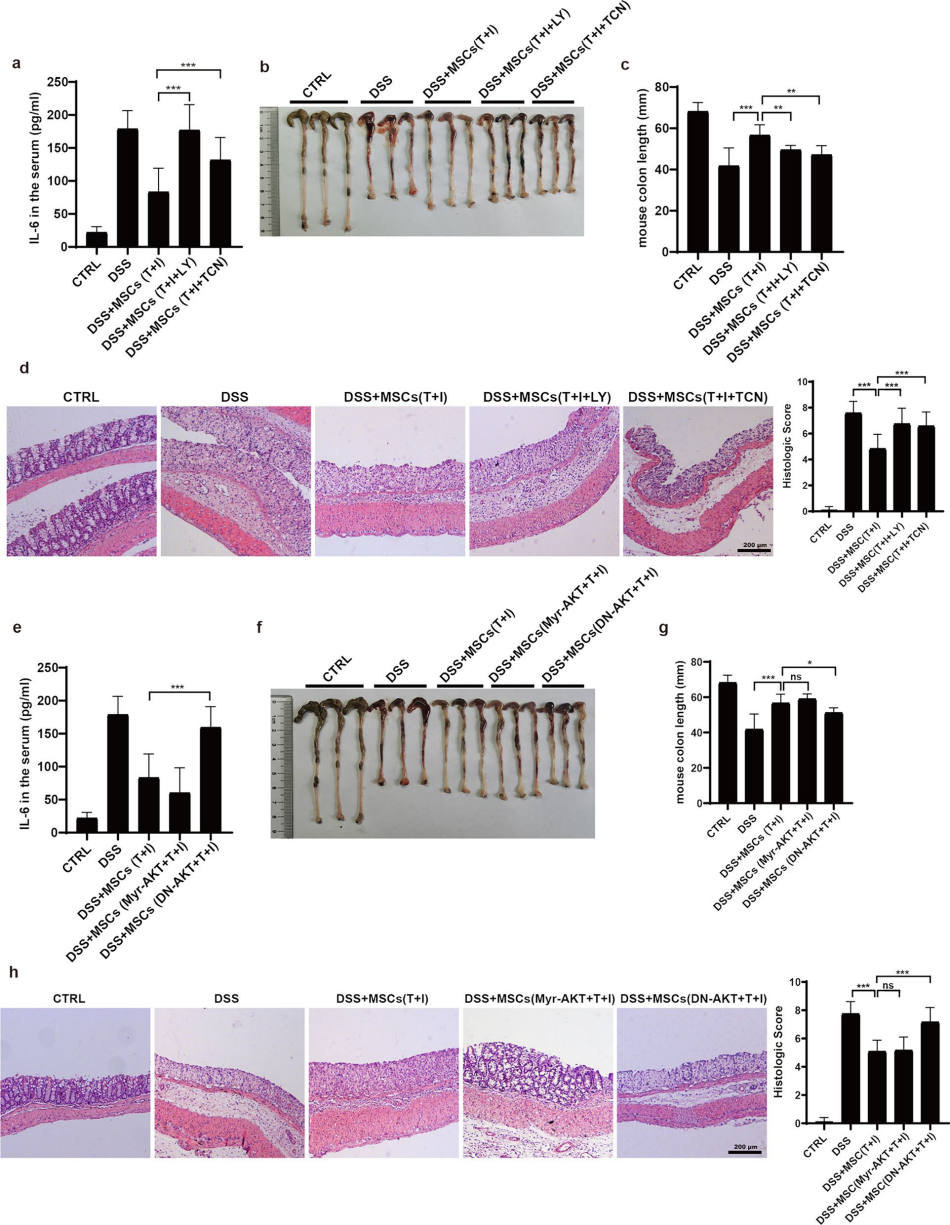
TNF*α*- and IFN*γ*-activated PI3K-AKT is required for the therapeutic effect of hUC-MSCs on IBD. **a**, **e** Bodyweight change of IBD mice treated with hUC-MSCs. The hUC-MSCs were pretreated with TNF*α* and IFN*γ* (10 ng/ml each) for 24 h. To block the glycolysis or OXPHOS, The hUC-MSCs were treated with 2-Deoxy-D-glucose (2-DG) or oligomycin (OM). **b**, **f** The IL-6 protein levels in the mouse serum were analyzed by ELISA. **c**, **g** The colon length of IBD mice treated with hUC-MSCs. The right panel shows the statistic analysis of the colon length of IBD mice. **d**, **h** The representative H&E stained colon sections of IBD mice treated with the hUC-MSCs and their histological scores. Scale bar, 200 μm. Data are presented as mean ± SEM. **, *p* < 0.01, ***, *p* < 0.001. *n* = 5 for each groups

Given that PI3K-AKT signaling could enhance the uptake of glucose and boost HKII activity, we next investigated whether the PI3K-AKT signaling is indeed required for the therapeutic effects of MSCs in vivo. We employed a 4% DSS-induced inflammatory bowel diseases (IBD) model in C57BL/6 J mice. The MSCs were treated with TCN or LY2940002 before they were infused into the mice with IBD. The level of inflammatory factor IL-6 was decreased after MSC infusion, while inhibition of PI3K-AKT signaling abolished the therapeutic effect (Fig. 6a). MSCs treated with TCN or LY294002 failed to attenuate the shortening of the colon caused by IBD (Fig. 6b, c). Histological sections showed that the bowel wall thickening and colon crypt damage in inhibitor-treated MSCs groups were obviously worse than in control groups (Fig. 6d). AKT dominant-negative form of AKT (DN-AKT) confirmed the specific role of AKT in MSCs therapy (Fig. 6e–h). Due to the high level of glycolysis induced by TNF*α* and IFN*γ*, constitutive activated AKT has little additional effect on IBD compared to TNF*α* and IFN*γ* treated groups (Fig. 6e–h). Collectively, these data showed that acutely activated PI3K-AKT signaling mediates the enhanced therapeutic effect of MSCs that is conferred by TNF*α* and IFN*γ*.

## Discussion

Many studies show that the choice of metabolic programs determines the function of immune cells. For example, quiescent T cells are kept in a much lower rate of energetic consumption and biosynthesis than the activated ones [24–26], and most of their ATP was produced by oxidative phosphorylation (OXPHOS) [27]. Upon stimulation, CD8+ T cells will rapidly switch to a metabolic profile characterized by increased rates of glucose uptake, glycolysis, and biosynthesis [27]. Likewise, CD4+ T cells will differentiate to T_H_1, T_H_2, and T_H_17 which requires glycolysis to support their effector functions [28–30]. For dendritic cells (DCs) [18] and natural killer (NK) cells [31], glycolysis is also enhanced after the activation. On the other hand, blocking glycolysis by 2-DG, a glycolysis-specific inhibitor, can improve the immunosuppressive function of Tregs and maintain the immune tolerance [32–34]. Upon activation, macrophages will be polarized into two different phenotypes, M1-like macrophages with pro-inflammation properties and M2-like macrophages with anti-inflammation function [35–37]. Increased glycolysis is required for the function of IFN*γ* or TLR ligands induced M1-like macrophages [38, 39]. Accordingly, M2-like macrophages prefer OXPHOS to support the anti-inflammation process while glycolysis is not a requirement for M2 macrophage polarization [40, 41]. The function of these immune cells requires the metabolic switch to support their functions. It seems that all the immune responses by the pro-inflammatory cells depend on glycolytic metabolism.

While the immune functions of immune cells are closely associated with distinct metabolic pathways [42, 43], the metabolic requirements for the immunomodulatory function of MSCs remain to be fully elucidated. In this study, we demonstrated that glycolysis is required for the anti-inflammatory effect of MSCs on IBD. TNF*α* and IFN*γ* drive glycolysis by rapidly activating PI3K/AKT signaling. This knowledge should be of value in improving the efficiency of MSCs-based cell therapy.

**Fig. 7:**
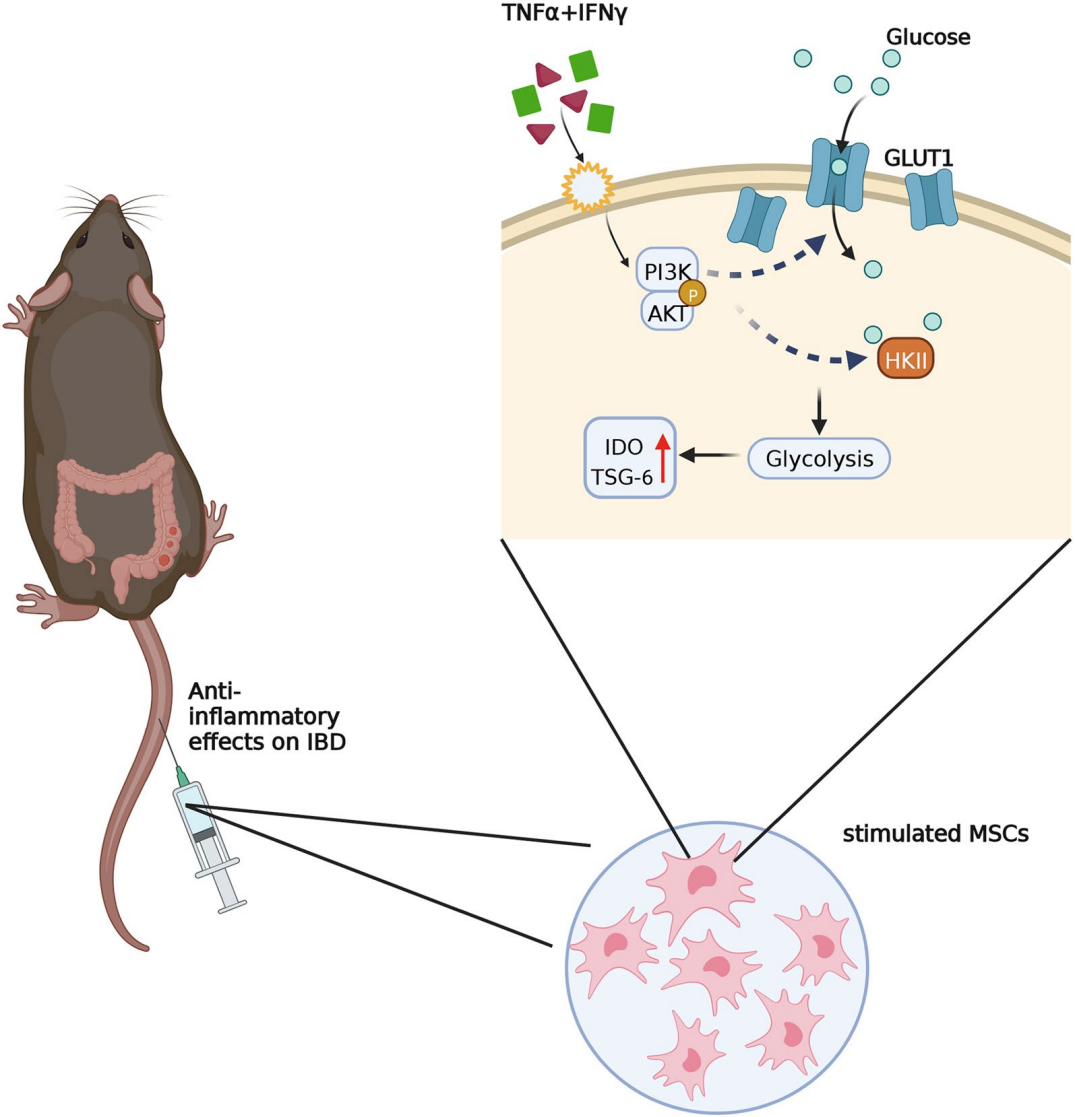
A schematic model of anti-inflammatory effects of MSCs on IBD mice. PI3K-AKT signaling regulates glycolysis in MSCs. PI3K-AKT signaling was activated rapidly leading to the increase of glucose uptake and HKII enzyme activity. Enhanced glycolysis in MSCs results in a more robust production of anti-inflammatory factors

On the basis of our findings, we propose that TNF*α* and IFN*γ* confer MSCs the enhanced immunomodulatory property by acutely activating PI3K/AKT signaling and consequently enhancing glycolysis (Fig. 7). Upon stimulation by TNF*α* and IFN*γ*, the MSCs respond rapidly. Phosphorylated AKT level is increased in 15 min. Blocking of PI3K enzyme activity by LY294002 inhibits glycolysis and impairs the expression of IDO1 and TSG-6. The AKT-specific inhibitor triciribine (TCN) also impede the glycolytic process. In contrast, LY294002 increased pAKT, which was also reported elsewhere [44]. We also monitored other signaling transduction, for example, ERK1/2 signaling. However, the activation status of ERK1/2 remained unchanged upon TNF*α* and IFN*γ* stimulation (data not shown). Taken together, these results indicate that PI3K/AKT axis participates in the regulation of glycolysis in MSCs.

The fact that TNF*α* and IFN*γ* acutely activated PI3K-AKT signaling (within 15 min) suggests that it is directly activated by TNF*α* and IFN*γ* through membrane receptors. Two specific TNF*α* receptors have been discovered. TNFR1 (p55) is expressed almost ubiquitously, while TNFR2 (p75) is mainly expressed in immune cells. TNF*α* binds to TNFR1 and activates three main signaling pathways including NF-*κ*B-signaling, MAPK/C-Jun-signaling, and caspase/apoptotic signaling [45, 46]. TNFR2 was reported to only respond to the membrane-bound form of TNF*α* and activate downstream PI3K/AKT [47]. Moreover, TNFR2 was also expressed in MSCs. Our data suggest that TNF*α* could directly activate downstream PI3K/AKT signaling in MSCs. In tumor cells, PI3K-AKT is involved in the IFN-*γ* induced phosphorylation of STAT1 (Ser-727) [44, 48, 49]. In cord blood-derived MSCs, PI3Ka mediates the IFN*γ*-induced STAT1 phosphorylation and IDO production [17]. But in our study, the pSTAT1 level in MSCs was not affected by LY294002.

After TNF*α* and IFN*γ* stimulation, the expression of IDO1 and TSG-6 protein increased tens of thousands of folds. Catabolic metabolism, especially glycolysis, provides the resource for gene transcription and translation. Protein biosynthesis needs a great amount of carbon skeleton and intermediates [20]. The glycolysis starting from the hydrolysis of glucose generates diverse carbon intermediates. In cancer cells, PI3K activation enhances glucose uptake and stimulate phosphofructokinase activity through the early parts of glycolysis which support macromolecular synthesis [50, 51]. Here, in the MSCs inflammation factors, TNF*α*- and IFN*γ*-induced glycolysis appeared to promote protein synthesis through the PI3K-AKT axis. Without the enhanced glycolysis, anti-inflammation factors IDO1 and TSG-6 could not be produced efficiently.

Future studies should investigate the exact mechanism whereby the PI3K-AKT pathway regulates the early steps of glycolysis in MSCs. The activation of PI3K-AKT signaling is a rapid but not sustainable signal to confer the anti-inflammation capability to MSCs. How to maintain this capability of MSCs is an important issue for future research.

## Conclusion

Our studies show that TNF*α* and IFN*γ* rapidly activate PI3K-AKT signaling and promote glycolysis in human UC-MSCs. Acutely activated PI3K-AKT drives the glucose uptake and HKII enzyme activity, which is responsible for the MSCs-based anti-inflammatory therapy. This knowledge could be used in the design of strategies to improve the anti-inflammation efficiency of MSCs in clinical situations.

## Supplementary Information

**Additional file 1. Table S1. **The primers used for real-time PCR.

## Data Availability

The datasets used and/or analyzed during the current study are available from the corresponding author on reasonable request.

## Abbreviations

MSCs: Mesenchymal stem cells
TNF-*α*: Tumor necrosis factor-*α*
IFN-*γ*: Interferon-*γ*
TSG-6: TNF-stimulated gene 6
IBD: Inflammatory bowel disease
DSS: Dextran sulfate sodium
ELISA: Enzyme-linked immunosorbent assay
IDO: Indoleamine 2,3-dioxygenase
OXPHOS: Oxidative phosphorylation
2-DG: 2-Deoxy-glucose
GvHD: Graft versus host disease
IL-1: Interleukin-1
ALI: Acute lung injury
PGE2: Prostaglandin E2
LPS: Lipopolysaccharide
PMSF: Phenylmethanesulfonyl fluoride
COX2: Cyclooxygenase-2
GLUT1: Glucose transporter 1
GAPDH: Glyceraldehyde-3-phosphate dehydrogenase
EAE: Experimental autoimmune encephalomyelitis
PI3K: Phosphoinositide 3-kinases
HRP: Horseradish peroxidase
TMB: 3,3′,5,5′-Tetramethylbenzidine
ECAR: Extracellular acidification
OM: Oligomycin
LY: LY294002
TCN: Triciribine
HA: Hemagglutinin
HLA-DR: Human leukocyte antigen-DR
CXCL9: C-X-C motif chemokine ligand 9
CXCL10: C-X-C motif chemokine ligand 10
CXCL11: C-X-C motif chemokine ligand 11
TLR: Toll-like receptor
2-NBDG: 2-(N-(7-nitrobenz-2-oxa-1,3-diazol-4-yl)amino)-2-deoxyglucose
HKII: Hexokinase II
DCs: Dendritic cells
NK cells: Natural killer cells
ERK: Extracellular signal-regulated kinase
ATP: Adenosine triphosphate
TNFR1: Tumor necrosis factor receptor 1
STAT1: Signal transducer and activator of transcription 1
MAPK: Mitogen-activated protein kinase
NF-*κ*B: Nuclear factor kappa-B
