# Supplementary material for: TNFα and IFNγ rapidly activate PI3K-AKT signaling to drive glycolysis that confers mesenchymal stem cells enhanced anti-inflammatory property
Source: Stem Cell Res Ther. 2022 Oct 4;13:491. doi: 10.1186/s13287-022-03178-3 (PMC9531381; doi:10.1186/s13287-022-03178-3)
Supplement: Supplementary file 1 — Additional file 1. Table S1. The primers used for real-time PCR. [file 13287_2022_3178_MOESM1_ESM.docx]

Table S1 The primers used for real-time PCR.

| Genes name (Human) | Oligonucleotide sequence (5′-3′) |
| --- | --- |
| β-actin | F: TTGCCGACAGGATGCAGAAGGA  R: GGTGGACAGCGAGGCCAGGAT |
| CXCL9 | F: CCAGTAGTGAGAAAGGGTCGC  R: AGGGCTTGGGGCAAATTGTT |
| CXCL10 | F: GTGGCATTCAAGGAGTACCTC  R: TGATGGCCTTCGATTCTGGATT |
| CXCL11 | F: GACGCTGTCTTTGCATAGGC  R: GGATTTAGGCATCGTTGTCCTTT |
| IDO1 | F: GCCCTTCAAGTGTTTCACCAA  R: CCAGCCAGACAAATATATGCGA |
| TSG-6 | F: TGTCTGTGCTGCTGGATGGAT  R: TGTGGGTTGTAGCAATAGGCAT |

Note: F, forward; R, reverse
